# Collective Victimhood and Ingroup Identity Jointly Shape Intergroup Relations, Even in a Non-violent Conflict: The Case of the Belgians

**DOI:** 10.5334/pb.334

**Published:** 2017-11-21

**Authors:** Alba Jasini, Ellen Delvaux, Batja Mesquita

**Affiliations:** 1 University of Leuven, BE

**Keywords:** collective victimhood, ingroup identity, intergroup emotions, action tendencies, non-violent conflict

## Abstract

Collective victimhood is the belief that one’s own group has been intentionally and undeservingly harmed by another group (Bar-Tal, Chernyak-Hai, Schori, & Gundar, 2009). While previous research has established the link between collective victimhood and negative intergroup behaviors, the underlying mechanism is virtually unexplored. In the current study, we test the idea that intergroup emotions play an important role, particularly for those group members who are highly identified. Whereas previous research has primarily studied collective victimhood in violent contexts, the current study focuses on its role in the intergroup relations in Belgium, known as a non-violent conflict between French and Dutch speakers.

The associations between collective victimhood, intergroup emotions, and action tendencies were studied in an online survey. The sample consisted of both French-speaking and Dutch-speaking Belgians (*N_total_* = 1774). Structural equation modeling showed that collective victimhood was negatively related to intergroup affiliative emotions (i.e., sympathy) and positively to intergroup distancing emotions (i.e., anger). In addition, these relationships were stronger for participants who strongly identified with their ingroup. Furthermore, intergroup affiliative emotions positively predicted fostering contact with outgroup members, and negatively predicted the tendencies to exclude and take revenge on the outgroup; intergroup distancing emotions positively predicted outgroup exclusion and revenge, and negatively predicted fostering contact with them. The established associations were no different between the linguistic groups. Our results confirm that collective victimhood, and the emotions associated, can help to understand intergroup conflict in non-violent contexts, in addition to violent ones.

*Collective victimhood* – the belief that one’s own group has been intentionally and undeservingly harmed by another group (Bar-Tal, Chernyak-Hai, Schori, & Gundar, 2009; Vollhardt, 2012) – is known to intensify negative intergroup behaviors. In violent conflicts, such as those in Northern-Ireland, Rwanda or Kosovo, perceived collective victimhood has been associated with mistrust of outgroup members, failure to forgive them for their past wrongdoings, and exclusion, which in turn are likely to contribute to the negative spiral of intergroup conflict (Andrighetto, Mari, Volpato, & Behluli, 2012; Noor, Brown, Gonzalez, Manzi, & Lewis, 2008; Vollhardt & Bilali, 2015). Whereas collective victimhood in violent conflicts has been extensively documented, little is known about its role in less or non-violent conflicts. The current research fills this gap by studying collective victimhood in the context of the Belgian linguistic conflict. The linguistic conflict in Belgium has been largely non-violent (Hooghe, 2004; Mnookin & Verbeke, 2009), and is fueled by collective memories of injustice to the Flemish, as well as current perceptions of injustices on both sides.

While we know that collective victimhood is linked to negative intergroup behaviors, the underlying mechanism is virtually unexplored (Noor, Brown, & Prentice, 2008). In this research, we investigate the role of intergroup emotions. Building on Intergroup Emotions Theory (IET, Mackie, Devos, & Smith, 2000), we propose that the perception of collective victimhood invokes intergroup emotions, which in turn prompt corresponding intergroup behaviors (Noor, Brown, & Prentice, 2008; Smith, Seger, & Mackie, 2007; Tam et al., 2007). Thus, previous research has borne out that individuals who perceived their group to be the victim of bad treatment by the outgroup, will experience negative emotions on behalf of the group (Noor, Brown, & Prentice, 2008). We expect to replicate this finding, and we predict that group-based emotions will prompt behavior that advances the interests of the in-group; this may involve harming the outgroup or preventing it from doing more harm to the ingroup (Bar-Tal et al., 2009; Mackie et al., 2000).

A final contribution of this research is to describe the role of individuals’ group identification. Intergroup Emotions Theory postulates that intergroup emotions are stronger the more group members identify with their group (Smith et al., 2007). If this were to apply in this context, we would expect that collective victimhood is particularly relevant (and emotion-evoking) for those individuals who are most identified with the ingroup. Therefore, we test the prediction that collective victimhood is particularly significant to people who are highly identified with one side of the conflict.

## Collective Victimhood in Violent and Non-violent Conflicts

Shared perceptions of collective victimhood play a role in the relations between the “victim”-group and the “perpetrator”-group. In violent intergroup contexts, collective victimhood has been found to motivate intergroup behavior. For instance, perceived collective victimhood comes with a decreased motivation for perspective taking, an increase in the extent to which group members infrahumanize the outgroup (Andrighetto et al., 2012), and higher levels of justification for aggression against the outgroup (Schori-Eyal, Halperin, & Bar-Tal, 2014). In addition, *competitive victimhood* – the belief that the ingroup has suffered *more* than the outgroup – makes group members less forgiving towards the outgroup (Noor, Brown, Gonzalez, et al., 2008); as well as less likely to own up to the ingroup responsibility for causing suffering on outgroup members (Cehajic & Brown, 2010). Finally, *exclusive victimhood* – the belief that the ingroup has suffered in unique ways that the other group has not –, is associated with taking more distance, mistrusting the outgroup more, and excluding the outgroup economically (Vollhardt & Bilali, 2015).

Most theorizing on collective victimhood has come from research in contexts of direct intergroup violence (Noor, Shnabel, Halabi, & Nadler, 2012; Vollhardt, 2012); victimhood in these contexts is very tangible in the form of death, physical injury and destruction (Galtung, 1969). Yet, beliefs of past victimization may also develop in contexts where direct intergroup violence is absent, but where there is inequality in the distribution of resources and power. In these cases, one of the groups is being unjustly disadvantaged, a phenomenon Galtung has referred to as ‘structural violence’ (Galtung, 1969). Structural violence consists of systematic impediments that prevent powerless individuals and groups in a society to meet their needs and achieve their potential (Galtung, 1969). Structural violence is usually embedded in the longstanding political, economic and social fabric of the society, and as a result it is anonymous or even legitimized over time (Farmer, 2004; Galtung, 1969). Yet, in societies that are characterized by a history of structural violence, individuals from powerless groups often suffer from poverty, and have poor access to education, health services, and employment (Farmer, 2004). At the level of the group, structural violence may harm social cohesion, harmony and integration into a society.

Very little is known about collective victimhood in “more peaceful” contexts (as noted by Noor et al., 2012). Improving our understanding of collective victimhood in those non-violent contexts would be useful in and of itself, but it would also provide a more dynamic understanding of collective victimhood in post-violent contexts. In many of these contexts, material and structural disadvantages survive the violence itself, and keep collective victimhood alive long after the violence has passed. A guiding question for this research will be how collective victimhood in non-violent conflict helps to instigate or maintain negative intergroup relations, and whether its effects are similar to the ones observed during violent conflict. We will investigate the role of (a) collective victimhood for emotions, and (b) emotions for action preparedness.

## Intergroup Emotions and Action Tendencies in Non-violent Conflicts

Collective victimhood is situated within an intergroup context, where members of one group perceive themselves as victims of the wrongdoings of another group. In this research, we propose that the significant role of collective victimhood in intergroup behavioral outcomes may be understood from the intergroup emotions that are related to beliefs of collective victimhood. According to Intergroup Emotions Theory, when members of a group appraise an event or situation in terms of their group membership, they will experience emotions on behalf of their ingroup. Intergroup emotions include action tendencies (Frijda, 1986, 2007) – i.e., motivations to act in ways that protect ingroup concerns (Frijda, 1986; Mackie et al., 2000) – and thus link beliefs of collective victimhood to action.

Emotions can be classified based on the ways the associated action tendencies affect the relationship with the outgroup. Prior work has distinguished between socially affiliative emotions and socially distancing emotions (Fischer & Manstead, 2008, 2016; Kitayama, Markus, & Kurokawa, 2000; Kitayama, Mesquita, & Karasawa, 2006; Mesquita, Marinetti, & Delvaux, 2012). Socially affiliative emotions (e.g., admiration, gratitude, shame) contribute to the maintenance and strengthening of the relationship with another person or group (Cuddy, Fiske, & Glick, 2008; Fischer & Manstead, 2008, 2016; Sweetman, Spears, Livingstone, & Manstead, 2013); in the intergroup context, they would be associated with action tendencies that serve the intentions for affiliating with the outgroup, such as the resolve to forget the past wrongdoings, forgive the outgroup, or engage in intergroup contact (Cehajic, Brown, & Castano, 2008; Noor, Brown, Gonzalez, et al., 2008). In contrast, socially distancing emotions (e.g., anger, contempt, pride) serve to distance oneself from, or cut all bonds with another person or group (Fischer & Manstead, 2008, 2016; Fischer & Roseman, 2007; Roseman, Wiest, & Swartz, 1994; Mesquita et al., 2012). In an intergroup context, they would be associated with action tendencies that aim to hurt or avoid the outgroup (Cuddy et al., 2008; Mackie et al., 2000). Previous research has indeed found an association between distancing emotions and the desire to attack (Maitner, Mackie, & Smith, 2006), take revenge (Halperin, Canetti, & Kimhi, 2012; Jasini & Fischer, 2017), or exclude the outgroup (Jasini & Fischer, 2017).

Only a few studies on collective victimhood have focused on emotion-related concepts. Previous studies with Protestant and Catholic groups in Northern Ireland, and pro- and anti-Pinochet groups in Chile, respectively, have found that perceptions of collective victimhood were negatively related with empathy and trust towards the outgroup (Noor, Brown, Gonzalez, et al., 2008; Noor, Brown, & Prentice, 2008). Relatedly, Dutch-speaking Belgians who highly trusted the outgroup that has victimized them in the past (French-speaking Belgians) reported more positive affiliative emotions (i.e., sympathy) and favorable attitudes towards the outgroup than those who showed less trust (Alarcón-Henríquez et al., 2010). Together, these studies suggest that collective victimhood comes with reduced affiliative emotions towards the outgroup. Given the associated perceptions of one’s ingroup being (intentionally) disadvantaged and harmed by the outgroup, we expect collective victimhood to come with increased distancing emotions as well.

In conclusion, we predict that the perception of collective victimhood is associated with decreases in affiliative emotions and increases in distancing emotions. In the current study, we focus on the intergroup emotions that serve an affiliative function towards the outgroup (e.g., sympathy), and intergroup emotions that are thought to serve a distancing function (e.g., anger). Previous research has shown that these emotions (a) are commonly experienced in intergroup contexts (Mackie et al., 2000; Van Acker, 2012), (b) are relevant in the face of intergroup inequality and injustice, and (c) fuel important intergroup behavior that determines harmonious, or conversely, disruptive intergroup relations. For instance, sympathy has emerged as an emotion that individuals experience on behalf of their group when they perceive the disadvantage of the outgroup as illegitimate (Harth, Kessler, & Leach, 2008; Iyer, Leach, & Crosby, 2003) and is therefore associated with behavioral tendencies that aim to help and support the disadvantaged outgroup (Harth et al., 2008; Iyer et al., 2003). In contrast, anger has been found to relate to aggressive and retaliatory tendencies (Doosje, Jonas, Jasini, Sveinsdóttir, & Erbas, 2016; Lickel, 2012) and to a desire to exclude and avoid the outgroup (Cuddy et al., 2008). Taken into account that beliefs of ingroup’s collective victimhood focus on the perception that the ingroup is in a disadvantaged position, and also limits perspective-taking of persons to see things form the standpoint of the other group (Andrighetto et al., 2012), we expect collective victimhood to be negatively associated with affiliative emotions targeted at the outgroup, and positively associated with distancing emotions targeted at the outgroup.

Finally, we will examine the associations of these different types of emotions with three groups of action tendencies that shape the relationship with the outgroup: revenge, exclusion and fostering contact. In a non-violent conflict context, revenge may consist of economic or political sanctions against the outgroup, and exclusion may manifest itself as an attempt to maintain distance, or ignore the outgroup’s existence. In contrast, fostering contact may consist of efforts to establish positive contact with the outgroup.

### Group Identification and Intergroup Emotions

Not every group member will have intergroup emotions to the same extent: We expect highly identified group members to have stronger intergroup emotions. This is the case, because the ingroup is more self-relevant for high than for low group-identifiers (Iyer & Leach, 2009). For instance, experimental lab studies have found that group members experience stronger emotions on behalf of the ingroup when their ingroup identity is made salient. Moreover, ingroup salience is particularly effective for individuals who identify strongly with the ingroup (Gordijn, Yzerbyt, Wigboldus, & Dumont, 2006; Yzerbyt, Dumont, Wigboldus, & Gordijn, 2003). For instance in a study in which undergraduate students were made to believe that they shared similarities with either a victim or a perpetrator group in an invented conflict between students and decision-making institutions, group members who categorized themselves in the same group as a victimized outgroup experienced more anger on behalf of this group; this effect was intensified for those who identified more strongly with the group (Gordijn et al., 2006). Following Intergroup Emotions Theory, we thus expect that ingroup identification predicts the level of intergroup emotions in contexts that are relevant to the interests of their ingroup.

## The Current Research

In sum, the aim of the current research was threefold: (1) to examine the role of collective victimhood in a non-violent conflict; (2) to investigate the mediating role of intergroup emotions in the association between collective victimhood and behavioral tendencies towards the “perpetrating” outgroup; and (3) to examine whether ingroup identification intensifies the impact of collective victimhood on intergroup emotions. To address these objectives, we made use of a large survey study in Belgium.

We focus on the Belgian context, because it is a context of non-violent intergroup conflict (Mnookin & Verbeke, 2009): There is both an economic and political conflict between Dutch-speaking and French-speaking Belgians, which started in 1830 when Belgium was founded, and the Dutch-speaking Northerners were dominated by the French-speaking Southerners. The collective disadvantage of the Dutch-speaking Belgians lasted until the mid-twentieth century. Since then, the power imbalance has shifted in favor of the Northern region, which is now both more prosperous and more powerful than the Southern region. Yet, many Dutch-speaking Belgians have strong collective memories of victimhood (Alarcón-Henríquez et al., 2010; Klein, Licata, Van der Linden, Mercy, & Luminet, 2012; Rimé, Bouchat, Klein, & Licata, 2015). At the same time, and as a consequence of their lost dominance, French-speaking Belgians currently feel collectively disadvantaged by the dominant Dutch-speaking group (Alarcón-Henríquez et al., 2010; Klein et al., 2012). Thus, both groups perceive being victimized by each other (Klein et al., 2012).

Our study includes data from both Dutch-speaking and French-speaking Belgians. We tested the following hypotheses in both groups:

H1a: Collective victimhood is negatively related to intergroup affiliative emotions.
H1b: Collective victimhood is positively related to intergroup distancing emotions.
H2a: Intergroup affiliative emotions are negatively related to exclusion and revenge.
H2b: Intergroup affiliative emotions are positively related to fostering contact.
H3a: Intergroup distancing emotions are positively related to exclusion and revenge.
H3b: Intergroup distancing emotions are negatively related to fostering contact.
H4: Intergroup emotions mediate the relationship between collective victimhood and action tendencies towards outgroup members.
H5: The relationship between collective victimhood and intergroup emotions is stronger for high compared to low ingroup identifiers.

## Method

### Participants

In May 2014, 1910 participants filled out an online questionnaire on the relationships between the Dutch- and French-speaking communities in Belgium. Considering participants’ answers on questions about their mother tongue and citizenship, we followed a case deletion procedure. Of the total sample, we excluded 136 participants: 27 participants were of non-Belgian origin, 17 participants had another mother tongue than Dutch or French, 28 participants failed to report their mother tongue, and 64 participants indicated to be bilingual, suggesting that they may identify with both groups simultaneously. The final sample of this study thus consisted of 1774 participants. They were on average 46 years old (*SD* = 17.23), and 62.5% of them were men (*n* = 1111). Based on the information on the mother tongue, 70% of the participants were categorized as French-speaking (*n* = 1244) and 30% as Dutch-speaking (*n* = 530).

### Procedure

This study was part of a larger research project on the relationships between Dutch- and French-speaking communities in Belgium, that was the result of a collaboration between three universities in Belgium (two universities based in the French-speaking part of Belgium, and one university based in the Dutch-speaking part of Belgium). The questionnaire was designed with the cooperation of researchers from all three universities. The items for the questionnaire were first designed in French, except for the questions on intergroup emotions and revenge tendency, which were first designed in Dutch. The French items were translated by native Dutch-speaking researchers, and the Dutch items were translated by native French-speaking researchers. After the translations were made, researchers of all teams checked the translations again in both languages.

The questionnaire was launched on May 5, and participants could fill out the questionnaire until May 25. Participants were recruited via the networks of the researchers, addressing family, friends, and students, and also via a publication on the website of daily newspapers as well as on the website of the different universities.

### Measures

Participants rated items measuring different constructs on a 7-point Likert scale, all ranging from 1 = *‘Totally disagree’* to 7 = *‘Totally agree’*, unless otherwise noted. Table 1 gives the means, standard deviations and reliabilities of each of the constructs, for French- and Dutch-speaking Belgians separately.

**Table 1:** Means, standard deviations and reliabilities of all variables of interest.

	*M* (F)	*M* (D)	*SD* (F)	*SD* (D)	Reliability (F)	Reliability (D)

Collective victimhood	2.98	4.97	1.68	1.83	–	–
Ingroup identification	3.83	4.38	1.92	1.78	.86	.82
Intergroup affiliative emotions	4.08	3.82	1.42	1.41	.80	.85
Intergroup distancing emotions	1.67	1.66	0.95	0.94	.81	.83
Exclusion	2.05	1.96	1.25	1.17	.81	.82
Revenge	1.59	1.66	1.19	1.34	.83	.89
Fostering contact	4.97	4.29	1.50	1.46	.81	.78

*Note.* F stands for French-speaking Belgians and D for Dutch-speaking Belgians. Reliabilities are based on Cronbach’s alphas for scales with more than two items, but on Spearman-Brown correlations for two-item scales (i.e., ingroup identification, revenge; Eisinga, Grotenhuis, & Pelzer, 2013).

*Collective victimhood*. Collective victimhood was measured with one item *(“Historically, Dutch- (French-)speaking Belgians suffered from the behavior of French- (Dutch-)speaking Belgians.”)*.

*Ingroup identification*. Ingroup identification was measured with two items (i.e., *“I am proud to tell my friends that I am Dutch- (French-)speaking.”; “Usually, I like to think of myself as a Dutch- (French-)speaker.”*).

*Intergroup emotions*. Intergroup emotions were measured with eight items (e.g., *anger, respect*). Participants were asked how strongly they felt each of these emotions towards outgroup members on a scale from 1 = *‘Not at all’* to 7 = *‘Very strong’*. To compute the scales, we conducted factor analyses separately for each linguistic group. The results were comparable for both groups. Thus, a factor analysis (Extraction Method: Principal Component Analysis; Rotation Method: Oblimin with Kaiser Normalization) on these items, which were used in previous research on intergroup relations (e.g., Cuddy et al., 2008; Smith et al., 2007; Van Acker, 2012) yielded two factors, namely intergroup affiliative emotions (3 items: *admiration, respect, sympathy*) and intergroup distancing emotions (5 items: *anger, frustration, resentment, contempt, aversion*). The affiliative emotions factor explained 22.22% of the total variance in the responses of the French-speaking group and 21.90% of the total variance in the Dutch-speaking group. The distancing emotions factor explained 42.64% of the total variance in the responses of the French-speaking group and 46.52% of the total variance in the Dutch-speaking group.

*Action tendencies*. To compute the action tendencies scales, we conducted factor analyses separately for each linguistic group. The results were comparable. Thus, a factor analysis (Extraction Method: Principal Component Analysis; Rotation Method: Oblimin with Kaiser Normalization) on 12 items, which were based on an adaptation of a forgiveness scale (Wade, 1989) and an adaption of a revenge scale (Jasini & Fischer, 2017), yielded three factors, namely exclusion (5 items: *“I have cut all ties with them.”; “I do not have any desire to have contact with them.”; “I am scared of situations that may bring me in contact with them.”; “I don’t trust them at all.”; “I organize my life pretending they don’t exist.“*), revenge (2 items: *“I would like them to experience the same injustice they have inflicted on us.”; “The only way that we can forget what we have gone through is if they have to go through the same thing themselves.“*) and fostering contact (4 items: *“I want to forget the past and instead concentrate on the future of our relationship.”; “I want to give them another chance and start our relationship with a clean slate.”; “I do everything in my power to make our relationship friendly again.”; “I think that it is possible to live with them in peace”*). The 12^th^ item *“I would like to show them how to treat us better“* was not retained based on the scale reliability analyses, which indicated a better reliability of the revenge scale if this item would be excluded. The exclusion factor explained 38.87% of the variance in the responses of the French-speaking group and 40.72% in the Dutch-speaking group. The revenge factor explained 9.75% of the variance in the French-speaking group and 9.37% in the Dutch-speaking group. The fostering contact factor explained 14.70% of the variance in the French-speaking group and 14.31% in the Dutch-speaking group.

An overview of the correlations between the variables of interest for both Dutch- and French-speaking Belgians can be found in Table 2.

**Table 2:** Correlations between all variables of interest.

	1	2	3	4	5	6	7

1. Collective victimhood	…	.39	–.28	.35	.40	.37	–.28
2. Ingroup identification	.21	…	–.20	.32	.30	.32	–.22
3. Intergroup affiliative emotions	–.24	–.13	…	–.34	–.53	–.36	.64
4. Intergroup distancing emotions	.32	.16	–.27	…	.49	.57	–.35
5. Exclusion	.34	.23	–.46	.51	…	.54	–.50
6. Revenge	.30	.21	–.29	.43	.50	…	–.34
7. Fostering contact	–.20	–.14	.56	–.29	–.47	–.33	…

*Note.* The correlations under the diagonal are found in the data of French-speaking Belgians and the correlations above the diagonal are found in the data of Dutch-speaking Belgians. All correlations are significant at the level *p* < .001.

### Analytic Strategy

To test our hypotheses, we used multi-group structural equation modeling. More specifically, we tested whether collective victimhood, ingroup identification and their interaction predicted intergroup affiliative and distancing emotions, and whether intergroup affiliative and distancing emotions predicted outgroup exclusion, revenge towards outgroup members, and fostering contact with outgroup members.

We assessed the goodness of fit of all models using the Chi-square statistic, the Root Mean Square Error of Approximation (RMSEA), the Standardized Root Mean Square Residual (SRMR) and the Comparative Fit Index (CFI). A model was considered as having acceptable to excellent fit if the RMSEA value was lower than 0.10 and preferably lower than 0.06, the SRMR was lower than 0.08 and preferably lower than 0.05, and the CFI value was higher than 0.90, and preferably higher than 0.95 (Hu & Bentler, 1999; Kline, 2005). Since the chi-square statistic is sensitive to large sample sizes, we expected the chi-square statistic to be significant in all the models and thus inappropriate for assessing the goodness of fit (Bentler & Bonett, 1980).

We first tested a model in which all paths of interest were freely estimated in both linguistic groups. To investigate whether the associations were similar across both groups, we then restricted the relationships between each pair of variables to be equal for both groups. Following common practice, we tested different models in an attempt to find the best trade-off between model fit and model complexity. To this aim, we estimated the change in model fit when going from more complex (but usually better fitting) models to less complex (but often worse fitting) models. We used the CFI and the RMSEA (Hu & Bentler, 1999; Kline, 2005) as indices for estimating changes in model fit. The more simple model was selected over the more complex model, if the change in CFI was smaller than or equal to –.010, and the change in RMSEA smaller than or equal to .015 (Cheung & Rensvold, 2002; Vandenberg & Lance, 2000).

To test whether collective victimhood and ingroup identification predicted action tendencies towards outgroup members via the intergroup emotions, in the final model, we estimated the indirect paths from collective victimhood and ingroup identification on the one hand and the intergroup tendencies on the other.

## Results

In our structural equation models, we investigated the relationships between collective victimhood/ingroup identification and intergroup emotions on the one hand and between intergroup emotions and tendencies towards outgroup members on the other. In addition, we tested whether the relationships were similar or different between the Dutch-speaking and the French-speaking Belgians. We first tested a model in which we specified all the hypothesized associations as follows: Collective victimhood, ingroup identification and the interaction between collective victimhood and identification (CV*ID) as predictors of the intergroup affiliative and distancing emotions, which in turn mediate the paths from the predictors to the intergroup tendencies of exclusion, revenge and fostering contact. In this model, we allowed for the paths in both linguistic groups to vary. The model showed an acceptable fit (*X^2^* (18, *N* = 1774) = 154.717, *p* < .001; RMSEA = .093; SRMR = .043; CFI = .956).

**Figure 1:**
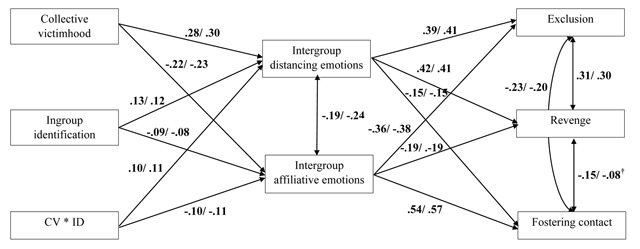
Multi-group structural equation model testing the relationship between collective victimhood and group identification, intergroup emotions, and action tendencies towards outgroup members. CV = Collective victimhood, ID = Ingroup identification. Path coefficients are standardized estimates. The first estimates are for the French-speaking group, the second estimates are for the Dutch-speaking group. All associations are statistically significant (*p* < .001) unless differently specified in the graph (*p* < .10).

In the following model, we tested whether the hypothesized associations were similar in both linguistic groups. We thus restricted the paths to be equal for both linguistic groups. This model showed a good fit (*X^2^* (30, *N* = 1774) = 192.362, *p* < .001; RMSEA = .078; SRMR = .049; CFI = .947). The very slight decrease in CFI (ΔCFI = –.009, which is below the conventional cut-off of –.010) and the decrease in RMSEA (ΔRMSEA = –0.015, which is below the conventional limit of .015), suggest that restriction of the model did not result in worse fit. Thus, all modeled paths were invariant between the two linguistic groups (see Figure 1 for the final model).

Consistent with our hypotheses, collective victimhood across linguistic groups was negatively related to intergroup affiliative emotions (Hypothesis 1a), and positively to intergroup distancing emotions (Hypothesis 1b). The more people perceived that their own linguistic group was (historically) harmed by the other linguistic group, the lower their intensity of affiliative emotions and the higher their intensity of distancing emotions towards members of the other linguistic group.

Also consistent with our predictions, intergroup affiliative emotions were found to negatively predict exclusion and revenge (Hypothesis 2a), and positively predict fostering contact with the outgroup (Hypothesis 2b). Furthermore, we confirmed that intergroup distancing emotions positively predicted exclusion and revenge (Hypothesis 3a), and negatively predicted fostering contact with the outgroup (Hypothesis 3b). Table 3 presents the explained variance for the different outcome variables by linguistic group.

**Table 3:** Explained variance (R^2^) of outcome variables by linguistic group.

	Estimate (Standard Error)	
	French-speaking group	Dutch-speaking group

Intergroup affiliative emotions	0.067 (.011)***	0.117 (.019)***
Intergroup distancing emotions	0.109 (.013)***	0.182 (.023)***
Exclusion	0.361 (.019)***	0.414 (.025)***
Revenge	0.259 (.018)***	0.258 (.023)***
Fostering contact	0.349 (.019)***	0.411 (.026)***

*Note.* ***p < .001.

To test if intergroup emotions mediate the associations between collective victimhood, and action tendencies (Hypothesis 4), we estimated the indirect effects in the model. Since the final model was equivalent in both groups, we tested the indirect paths across groups. In line with our predictions, all indirect effects were found to be statistically significant (see Table 4). Thus, intergroup affiliative emotions and intergroup distancing emotions significantly mediated the paths from collective victimhood to revenge, exclusion and fostering contact; and this was true for both Dutch-speaking and French-speaking Belgians.

**Table 4:** Mediation by intergroup emotions in the relationship between collective victimhood, ingroup identity and action tendencies.

	Estimate (standard error)		
Collective victimhood	Exclusion	Revenge	Fostering contact

via Intergroup affiliative emotions	0.058 (.008)***	0.031 (.005)***	–0.105 (.013)***
via Intergroup distancing emotions	0.080 (.009)***	0.084 (.011)***	–0.036 (.006)***
**Ingroup identity**	**Exclusion**	**Revenge**	**Fostering contact**

via Intergroup affiliative emotions	0.021 (.006)***	0.011 (.003)***	–0.037 (.011)***
via Intergroup distancing emotions	0.033 (.006)***	0.035 (.007)***	–0.015 (.004)***

*Note.* ***p < .001, **p < .01.

**Figure 2:**
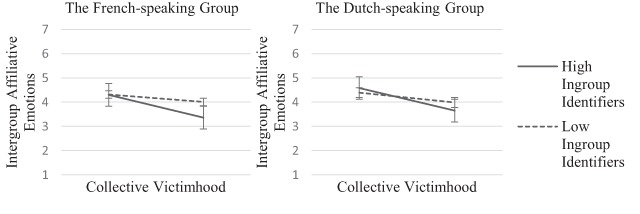
Intergroup affiliative emotions as a function of collective victimhood and ingroup identification.

**Figure 3:**
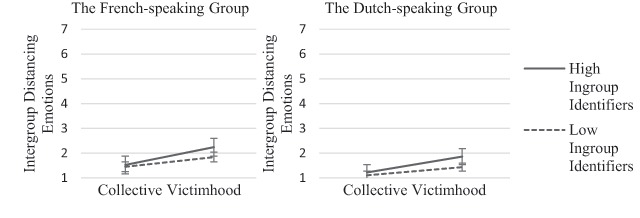
Intergroup distancing emotions as a function of collective victimhood and ingroup identification.

Finally, in line with our expectation that ingroup identification would alter the association between collective victimhood and the intergroup emotions and tendencies (H5), the model shows that individuals high on perceived collective victimhood and high on ingroup identification reported lower levels of intergroup affiliative emotions (*β*_CV*ID_ = –0.100, *SE*_CV*ID_ = .022, *p* < .001 for the French-speaking group, and *β*_CV*ID_ = –0.115, *SE*_CV*ID_ = .025, *p* < .001 for the Dutch-speaking group) and higher levels of intergroup distancing emotions (*β*_CV*ID_ = 0.096, *SE*_CV*ID_ = .021, *p* < .001 for the French-speaking group, and *β*_CV*ID_ = 0.112, *SE*_CV*ID_ = .024, *p* < .001 for the Dutch-speaking group) than any of the other groups, and this was true both for the Dutch-speaking and the French-speaking group (see Figures 2 and 3). In addition, we found that the ingroup identification alone was positively related to intergroup distancing emotions, and negatively to intergroup affiliative emotions. Thus, the more strongly participants identified with their linguistic group, the more intense distancing emotions and the less intense affiliative emotions for the outgroup they reported.

## Discussion

The current study aimed to investigate a context of non-violent intergroup conflict. We were specifically interested to learn i) whether intergroup emotions mediate the relationship between perceived collective victimhood and action tendencies towards outgroup members, and ii) whether ingroup identification moderates the relationship between perceptions of collective victimhood and intergroup emotions. Our study investigated these questions with Dutch-speaking and French-speaking Belgians, who have a history of non-violent conflict, in which both parties have reasons to perceive collective victimhood (Klein et al., 2012).

Our results show that across linguistic groups the perception of collective victimhood was associated with both intergroup affiliative emotions (e.g., sympathy, respect) and intergroup distancing emotions (e.g., anger, contempt). Moreover, these intergroup emotions mediated the relationship between collective victimhood and intergroup action tendencies. More specifically, the more strongly participants endorsed perceptions of collective victimhood, the less intense their intergroup affiliative emotions and the more intense their intergroup distancing emotions were. Intergroup affiliative emotions were in turn associated with a weaker tendency to exclude and to take revenge on the outgroup, and a stronger tendency to foster contact with the outgroup. The opposite pattern was found for the intergroup distancing emotions: they were associated with a stronger tendency to exclude and to take revenge on the outgroup, and a weaker tendency to foster contact with the outgroup. Furthermore, highly identified group members who perceived collective victimhood reported lower levels of intergroup affiliative emotions and higher levels of intergroup distancing emotions than other group members.

Our study extends previous knowledge on the role of collective victimhood in intergroup conflict in several ways. First, the current research suggests that collective victimhood is pertinent in non-violent contexts that are characterized by unbalanced power dynamics, and structural and institutional deprivation for which the outgroup is blamed. It also shows that group members may have a sense of collective victimhood because of the disadvantage in the collective past (as was the case for Dutch-speaking Belgians), or because of ongoing conflict (as was the case for the French-speaking Belgians in this study). Interestingly, the average levels of collective victimhood obtained in this study are comparable with the levels reported in studies that were carried out in violent conflict contexts (for instance, the Israel-Palestinian conflict, Schori-Eyal et al., 2014).

Second, our research shows that collective victimhood is associated with action tendencies through intergroup emotions. Previous research on collective victimhood has largely failed to acknowledge this important role of intergroup emotions.

Third, the current research highlights the role of ingroup identification, with highly identified group members having stronger intergroup emotions when they feel collectively victimized than less identified group members. It is important to note that differently from previous theorizing that emphasizes a strong relation between collective victimhood and ingroup identification (Andrighetto et al., 2012; Bar-Tal et al., 2009), in our study, we conceptualize ingroup identification as a factor that may intensify the emotional effects of the personal endorsement of a shared representation of the conflict.

Finally, the current research extends previous knowledge on the relations between the two main linguistic communities in Belgium, by showing that beliefs of past and current victimhood are real, and that they influence the relation between the linguistic groups. Perceptions of collective victimhood have been historically central to the Dutch-speaking community (Rimé et al., 2015), but are currently also pertinent to French-speaking Belgians who feel disadvantaged in the current context. This finding bears significance in light of previous theorizing that current events of injustice may be exploited to justify past narratives about the outgroup (Bar-Tal & Cehajic-Clancy, 2014).

## Limitations and future directions

The current study has some limitations. First, the sample was not representative of the Belgian population. French-speaking Belgians were overrepresented in the sample, possibly as a result of better advertising in French-speaking universities. However, the large sample size, and the replication of the major associations across the linguistic groups, inspires confidence in the established links between collective victimhood, ingroup identification, intergroup emotions, and behavioral tendencies.

Second, we investigated no more than a few socially affiliative and distancing emotions, and in this study, all affiliative emotions were positively valenced and all distancing emotions negatively. Therefore, the current study does not allow to test whether affiliation and distance, rather than valence, best describe the relevant emotional dimension in intergroup contact. However, we believe that the relational orientation of the emotion rather than its valence will determine outcomes in intergroup relations. For instance, we believe that ingroup pride, a positive group-based emotion, may have a socially distancing function towards the outgroup, whereas some negative emotions such as guilt, shame, or sadness may have a socially affiliating function towards the outgroup (Fischer & Manstead, 2016). Future research including other positive and negative emotions will need to test these hypotheses.

Third, the cross-sectional design of this study limits our understanding of the direction of the associations we found in our model. Future longitudinal research may help to shed light on the causal direction of associations between collective victimhood, ingroup identification, intergroup emotions and action tendencies over time. In a similar vein, future longitudinal research may want to examine how past victimhood perceptions fuel perceptions of current and future victimhood, which may contribute to the lingering negative attitudes and discriminatory behaviors towards the outgroup. In the current research, we explored whether perceptions of past, current and future victimhood were correlated with one another, and whether perceptions of current and future ingroup victimization were associated with intergroup emotions and tendencies in a similar way as the perceptions of past victimhood. The three measures of victimhood were moderately correlated. In addition, the perceptions of current and future ingroup victimhood were also correlated with intergroup emotions and action tendencies in similar ways as the past victimhood was. These exploratory findings suggest a long-lasting effect of past victimhood to the future, and perhaps an evaluation of the outgroup in this context as unchanging over time.

Fourth, it would be interesting to know if the victims of different kinds of violence would develop different kinds of emotions or action tendencies towards the outgroup. For instance, anger and sympathy-like emotions may be prevalent and relevant emotions in contexts of all possible types of intergroup violence but their predicting strength of the intergroup tendencies may be different. Thus, it may be possible that intergroup distancing emotions, such as anger and contempt, may induce more violent forms of revenge and exclusion in the context of direct violence.

Finally, the current findings speak to policy makers, and others who are interested in building harmonious intergroup relations. Our findings reveal that historical representations of collective victimhood in non-violent contexts have an important emotional aspect, and may serve as a barrier to conflict resolution and harmonious contact between members of the groups (Bar-Tal & Halperin, 2011). An important implication of this finding is that policy makers should pay close attention to the specific representations of past intergroup relations and their emotional and behavioral consequences when designing initiatives for increasing contact and reducing the discriminatory behaviors between groups. Future applied research may want to investigate whether practices commonly used in conflict-reconciliatory practices, such as creating a platform where sharing and discussing these representations in a constructive way, are feasible and effective in reducing negative intergroup outcomes in Belgium. In addition, as suggested by previous studies with Dutch-speaking Belgians (Alarcón-Henríquez et al., 2010), it may be worthwhile to investigate whether receiving recognition from the outgroup on the suffering it has caused in the past, may improve intergroup attitudes. Moreover, future research may also focus on how building a shared vision of harmonious and engaged intergroup relations may decrease the lingering effects of past victimhood beliefs.

To conclude, this study bridges research on collective victimhood and intergroup emotions by showing that intergroup emotions mediate the relationship between beliefs of collective victimhood and action tendencies towards the perpetrator outgroup. Moreover, the relationship between collective victimhood and intergroup emotions was found to be stronger for individuals who were highly identified with their groups than for those who were less identified.

## References

[1] Alarcón-Henríquez A., Licata L., Leys C., Van Der Linden N., Klein O., Mercy A. (2010). Recognition of shared past sufferings, trust and improving
intergroup attitudes in Belgium. Revista de Psicología.

[2] Andrighetto L., Mari S., Volpato C., Behluli B. (2012). Reducing competitive victimhood in Kosovo: The role of extended
contact and common ingroup identity. Political Psychology.

[3] Bar-Tal D., Cehajic-Clancy S., Spini D., Elcheroth G., Biruski D. C. (2014). From collective victimhood to social reconciliation: Outlining a
conceptual framework. War, community, and social change.

[4] Bar-Tal D., Chernyak-Hai L., Schori N., Gundar A. (2009). A sense of self-perceived collective victimhood in intractable
conflicts. International Review of the Red Cross.

[5] Bar-Tal D., Halperin E. (2011). Socio-psychological barriers to conflict
resolution. Intergroup Conflicts and Their Resolution: A Social Psychological
Perspective.

[6] Bentler P. M., Bonett D. G. (1980). Significance tests and goodness of fit in the analysis of
covariance structures. Psychological Bulletin.

[7] Cehajic S., Brown R. (2010). Silencing the past: Effects of intergroup contact on
acknowledgment of in-group. Social Psychological and Personality Science.

[8] Cehajic S., Brown R., Castano E. (2008). Forgive and forget? Consequences and antecedents of intergroup
forgiveness in Bosnia and Herzegovina. Political Psychology.

[9] Cheung G. W., Rensvold R. B. (2002). Evaluating goodness-of- fit indexes for testing measurement
invariance. Structural Equation Modeling: A Multidisciplinary Journal.

[10] Cuddy A. J. C., Fiske S. T., Glick P., Zanna M. P. (2008). Warmth and competence as universal dimensions of social
perception: The stereotype content model and the BIAS map. Advances in Experimental Social Psychology.

[11] Doosje B., Jonas K., Jasini A., Sveinsdóttir G., Erbas Y. (2016). When your nation has been disgraced: The experience of national
humiliation and its behavioral tendencies in dignity, honor and face-keeping
cultures.

[12] Eisinga R., te Grotenhuis M., Pelzer B. (2013). The reliability of a two-item scale: Pearson, Cronbach, or
Spearman-Brown?. International Journal of Public Health.

[13] Farmer P. (2004). An anthropology of structural violence. Current Anthropology.

[14] Fischer A. H., Manstead A. S. R., Lewis M., Haviland-Jones J. M., Barrett L. F. (2008). Social functions of emotion. Handbook of emotions.

[15] Fischer A. H., Manstead A. S. R., Barrett L. F., Lewis M., Haviland-Jones J. M. (2016). Social functions of emotion and emotion
regulation. Handbook of emotions.

[16] Fischer A. H., Roseman I. J. (2007). Beat them or ban them: The characteristics and social functions
of anger and contempt. Journal of Personality and Social Psychology.

[17] Frijda N. H. (1986). The emotions.

[18] Frijda N. H. (2007). The laws of emotion.

[19] Galtung J. (1969). Violence, peace, and peace research. Journal of Peace Research.

[20] Gordijn E. H., Yzerbyt V., Wigboldus D., Dumont M. (2006). Emotional reactions to harmful intergroup
behavior. European Journal of Social Psychology.

[21] Halperin E., Canetti D., Kimhi S. (2012). In love with hatred: Rethinking the role hatred plays in shaping
political behavior. Journal of Applied Social Psychology.

[22] Harth N. S., Kessler T., Leach C. W. (2008). Advantaged group’s emotional reactions to intergroup
inequality: The dynamics of pride, guilt, and sympathy. Personality and Social Psychology Bulletin.

[23] Hooghe L., Amoretti U. M., Bermeo N. (2004). Belgium: Hollowing the center. Federalism and territorial cleavages.

[24] Hu L., Bentler P. M. (1999). Cutoff criteria for fit indexes in covariance structure analysis:
Conventional criteria versus new alternatives. Structural Equation Modeling: A Multidisciplinary Journal.

[25] Iyer A., Leach C. W. (2009). Emotion in inter-group relations. European Review of Social Psychology.

[26] Iyer A., Leach C. W., Crosby F. J. (2003). White guilt and racial compensation: The benefits and limits of
self-focus. Personality and Social Psychology Bulletin.

[27] Jasini A., Fischer A. H. (2017). Characteristics and social determinants of intergroup hate.

[28] Kitayama S., Markus H. R., Kurokawa M. (2000). Culture, emotion, and well-being: Good feelings in Japan and the
United States. Cognition & Emotion.

[29] Kitayama S., Mesquita B., Karasawa M. (2006). Cultural affordances and emotional experience: Socially engaging
and disengaging emotions in Japan and the United States. Journal of Personality and Social Psychology.

[30] Klein O., Licata L., Van der Linden N., Mercy A., Luminet O. (2012). A waffle-shaped model for how realistic dimensions of the Belgian
conflict structure collective memories and stereotypes. Memory Studies.

[31] Kline R. B. (2005). Principles and practice of structural equation
modeling. Methodology in the Social Sciences.

[32] Lickel B., Tropp L. R. (2012). Retribution and revenge. The Oxford handbook of intergroup conflict.

[33] Mackie D. M., Devos T., Smith E. R. (2000). Intergroup emotions: Explaining offensive action tendencies in an
intergroup context. Journal of Personality and Social Psychology.

[34] Maitner A. T., Mackie D. M., Smith E. R. (2006). Evidence for the regulatory function of intergroup emotion:
Emotional consequences of implemented or impeded intergroup action
tendencies. Journal of Experimental Social Psychology.

[35] Mesquita B., Marinetti C., Delvaux E., Fiske S. T., McCrae C. N. (2012). The social psychology of emotions. Sage Handbook of social cognition.

[36] Mnookin R., Verbeke A. (2009). Persistent nonviolent conflict with no reconciliation: The
Flemish and Walloons in Belgium. Law and Contemporary Problems.

[37] Noor M., Brown R., Gonzalez R., Manzi J., Lewis C. A. (2008). On positive psychological outcomes: What helps groups with a
history of conflict to forgive and reconcile with each
other?. Personality and Social Psychology Bulletin.

[38] Noor M., Brown R., Prentice G. (2008). Precursors and mediators of intergroup reconciliation in Northern
Ireland: A new model. British Journal of Social Psychology.

[39] Noor M., Shnabel N., Halabi S., Nadler A. (2012). When suffering begets suffering: The psychology of competitive
victimhood between adversarial groups in violent conflicts. Personality and Social Psychology Review.

[40] Rimé B., Bouchat P., Klein O., Licata L. (2015). When collective memories of victimhood fade: Generational
evolution of intergroup attitudes and political aspirations in
Belgium. European Journal of Social Psychology.

[41] Roseman I. J., Wiest C., Swartz T. S. (1994). Phenomenology, behaviors, and goals differentiate discrete
emotions. Journal of Personality and Social Psychology.

[42] Schori-Eyal N., Halperin E., Bar-Tal D. (2014). Three layers of collective victimhood: Effects of multileveled
victimhood on intergroup conflicts in the Israeli-Arab
context. Journal of Applied Social Psychology.

[43] Smith E. R., Seger C. R., Mackie D. M. (2007). Can emotions be truly group level? Evidence regarding four
conceptual criteria. Journal of Personality and Social Psychology.

[44] Sweetman J., Spears R., Livingstone A. G., Manstead A. S. R. (2013). Admiration regulates social hierarchy: Antecedents, dispositions,
and effects on intergroup behavior. Journal of Experimental Social Psychology.

[45] Tam T., Hewstone M., Cairns E., Tausch N., Maio G. R., Kenworthy J. (2007). The impact of intergroup emotions on forgiveness in Northern
Ireland. Group Processes & Intergroup Relations.

[46] Van Acker K. (2012). Flanders’ real and present threat. How representations of
intergroup relations shape attitudes towards Muslim minorities. (Unpublished
doctoral dissertation).

[47] Vandenberg R. J., Lance C. E. (2000). A review and synthesis of the measurement invariance literature:
Suggestions, practices, and recommendations for organizational
research. Organizational Research Methods.

[48] Vollhardt J. R., Tropp L. R. (2012). Collective victimization. The Oxford Handbook of intergroup conflict.

[49] Vollhardt J. R., Bilali R. (2015). The role of inclusive and exclusive victim consciousness in
predicting intergroup attitudes: Findings from Rwanda, Burundi, and
DRC. Political Psychology.

[50] Wade S. H. (1989). The development of a scale to measure forgiveness. Unpublished doctoral
dissertation.

[51] Yzerbyt V., Dumont M., Wigboldus D., Gordijn E. (2003). I feel for us: The impact of categorization and identification on
emotions and action tendencies. The British Journal of Social Psychology/the British Psychological
Society.

